# Hesperidin exacerbates the therapeutic potency of cisplatin against hepatocytotoxicity of Ehrlich ascites carcinoma in mice

**DOI:** 10.1038/s41598-025-02442-9

**Published:** 2025-06-27

**Authors:** Nahed Saleh, Tamer Allam, Reda M. S. Korany, Abdelfattah M. Abdelfattah, Ahmed M. Omran, Mabrouk Attia Abd Eldaim, Nermeen Borai El-Borai

**Affiliations:** 1 Department of Clinical Pathology, Faculty of Veterinary Medicine, University of Sadat City, Sadat City, Menoufia 32897 Egypt; 2 Department of Pathology, Faculty of Veterinary Medicine, Cairo University, Giza, 12211 Egypt; 3 Department of Biochemistry and Molecular Biology, Faculty of Veterinary Medicine, Menoufia University, Sheben Elkom, 32511 Egypt; 4 Department of Biochemistry and Molecular Biology, Faculty of Veterinary Medicine, Menoufia National University, Birket El Sabaa, Menoufia Egypt; 5 Department of Forensic Medicine and Toxicology, Faculty of Veterinary Medicine, University of Sadat City, Sadat City, Menoufia 32897 Egypt

**Keywords:** Cisplatin, Ehrlich ascites carcinoma, Hesperidin, Alpha fetoprotein, Caspase-3, Ki-67, Biochemistry, Cancer, Drug discovery, Biomarkers, Medical research

## Abstract

The present research was set out to delineate the protective and therapeutic potency of hesperidin (Hesp) versus cisplatin (Cis) against the deleterious consequences of Ehrlich ascites carcinoma (EAC) on the liver and the prospective mitigative effect of Hesp against Cis-mediated hepatotoxic side-effects. A total of 70 female mice were randomly assigned into control, Hesp, EAC, Hesp-protected, Hesp-treated, Cis-treated, and Cis + Hesp-treated groups. Mice inoculated with EAC cells exhibited significant reductions in the serum total protein and albumin levels, along with significant elevations of the serum aminotransferases, lactate dehydrogenase, amylase, and lipase activities, and alpha-fetoprotein level. A significant increment in malondialdehyde level concomitantly with significant declines in reduced glutathione concentration and catalase activity were also observed in the liver of EAC-bearing mice. Additionally, marked hepatic pathological changes as well as a strong Ki-67 expression and a weak caspase-3 expression in the neoplastic cells infiltrating hepatocytes were observed. In contrast, the administration of Hesp and/or Cis to the EAC-bearing mice reversed, to varying degrees, the cytotoxic effects of EAC. Besides, Hesp minimized the harmful hepatic chemotherapeutic side-effects of Cis. Overall, Hesp could be a promising phytochemical against EAC-induced cytotoxicity with its potential to improve the antitumor efficacy of chemotherapeutic drugs and minimize their hepatic adverse side-effects.

## Introduction

Nowadays, cancer is regarded as one of the most dreadful causes of mortality all over the world^1^. Cancer is the consequence of a complex multistep, multifocal, and multipath process that includes a series of cellular epigenetic and/or genetic alterations, resulting in genomic instability and cancer development and progression^2^.

Ehrlich ascites carcinoma (EAC) is an undifferentiated carcinoma similar to human tumors, which are distinguished by rapid proliferation, high translatable ability, short life span, 100% malignancy, and a lack of the tumor-specific transplantation antigen^3,4^. Resembling the other tumors in body cavities, EAC cells rapidly developed in the peritoneal cavity due to the rapid cell division that cause a local inflammatory response, along with the increase of vascular permeability and accumulation of hemorrhagic ascitic fluid in the peritoneal cavity, where the cells rapidly proliferate and then migrate into the internal organs^5,6^, resulting in various adverse effects to numerous organs, including liver, kidney and heart^7–9^.

Among the chemotherapeutic medications, cisplatin (Cis) is the most widely used for the treatment of various malignancies^10^. Its mode of action has been linked to its ability to crosslink with the purine bases on the DNA; disrupting DNA repair mechanisms, inducing DNA damage, and ultimately triggering apoptosis in cancer cells^11^.

Despite its anticancer efficacy, the use of Cis as anticancer agent is currently limited because of the development of drug resistance to cancer cells that has been reported in numerous in vitro and in vivo investigations, in addition to its adverse side effects, particularly on the liver and kidney^9,11,12^. As a result, combinatorial therapies may be helpful in overcoming Cis-resistance and limiting its detrimental side-effects.

Considering the beneficial role in the food sector and human health, antioxidants are gaining popularity all over the world. Medicinal plants are easily available and potent source of natural antioxidants as they contain a mixture of phytochemicals, which possess numerous pharmacological properties^13^. Consequently, medicinal plants may be a promising alternative medicine to the chemotherapeutic drugs. Citrus fruits are among the most common medicinal plants that have attracted scientific interest because of their nutritional and health-promoting values, which is closely related to their high contents of numerous phytochemicals^14^. Hesperidin is the dominant flavanone glycoside originally found in citrus fruits such as lemons, clementine, grapefruit, mandarins, and oranges^15^. Numerous in vitro and in vivo literatures have proven the diverse pharmacological properties of Hesp, including antidiabetic, antihypertensive, anti-inflammatory, antioxidant, and hypolipidemic effects^9,16,17^. In addition, Hesp has a protective role against hepatic, renal, cardiovascular, and neurodegenerative disorders^9,16–19^. Recently, numerous studies proved the ability of Hesp to modulate the key hallmarks of cancer, including suppressing pro-inflammatory molecules, enhancing antioxidant defenses, and inhibiting cancer cell growth by promoting apoptosis, thereby exerting a potent anticarcinogenic effect^9,18,19^.

Based on this background, the objective of this study was to evaluate the protective and therapeutic effects of hesperidin, a natural phytochemical, compared to cisplatin, a common chemotherapeutic agent, against the deleterious consequences of Ehrlich ascites carcinoma on the liver, with particular emphasis on hesperidin’s potential to mitigate cisplatin-induced hepatotoxic side-effects.

## Materials and methods

### Chemicals

Cisplatin (Cisplatin® Mylan, Saint-Priest, France, 50 mg/50 mL vial) was purchased from a local pharmacy (Sadat City, Egypt). Hesperidin (≥ 80% purity) was obtained from Sigma-Aldrich Chemical Company (St. Louis, MO, USA). The diagnostic kit for assaying serum alpha-fetoprotein (AFP) level was obtained from Biomerieux Company (Marcy-L’Etoile, France), while those for assaying serum total protein, albumin, cholesterol, and glucose levels; alanine aminotransferase (ALT), aspartate aminotransferase (AST), and lactate dehydrogenase (LDH) activities, were received from Spectrum Diagnostic Company (Obour City, Egypt). Serum activities of amylase and lipase were measured using kits of Spinreact Diagnostic (Lab Supply co., Cairo, Egypt). Determination of malondialdehyde (MDA) level, reduced glutathione (GSH) concentration, and catalase (CAT) activity in liver tissues was performed using diagnostic kits supplied by Biodiagnostics Company (Dokki, Egypt). All other reagents or chemicals used were commercially available and of analytical grade.

### Experimental animals

Seventy female Swiss albino mice, weighing between 25 and 30 g, were received from the Animal Care Unit of Vacsera Pharmaceutical Company, Egypt. Mice were caged at a room with standard laboratory conditions (23 ± 2 °C; 45–50% RH; natural daily dark/light cycle) and provided tap water and clean food ad libitum all over the experimental period. The experimental design was ethically approved (Approval No. VUSC-003–1-21) by the Institutional Animal Care and Use Committee, Faculty of Veterinary Medicine, University of Sadat City, Egypt. The study follows the Guide for the Care and Use of Laboratory Animals 8th edition. Washington (DC): National Academies Press (US); 2011 and approved by IACUC of USC.

### Preparation and inoculation of EAC cells

The EAC cells were obtained by aspiration of 0.2 mL of ascetic fluid from the EAC-bearing mice, which were provided from the National Cancer Institute, Cairo University, Egypt. The aspirated fluid was diluted at a rate of 1:4 with sterile isotonic saline. Each mouse was intraperitoneally injected once with 2.5 × 10^6^ viable EAC cells/0.2 mL diluted ascetic fluid^8^.

### Experimental design

**Fig. 1:**
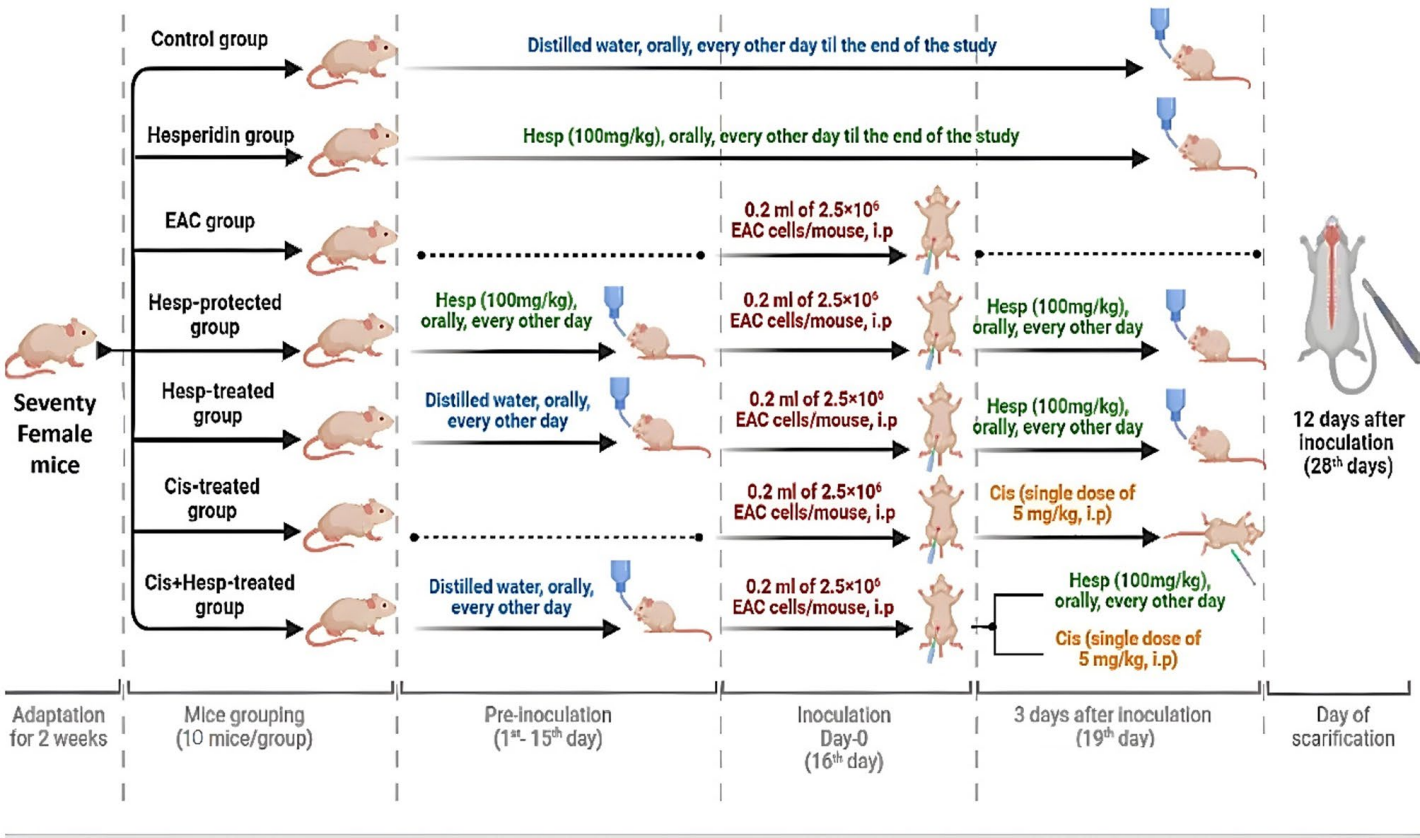
The experimental design and animal grouping. EAC: Ehrlich Ascites Carcinoma, Hesp: hesperidin, Cis: cisplatin.

Mice were indiscriminately allocated into 7 groups, each of 10 mice (Fig. 1).

*Control group*: Mice received distilled water orally, every other day, throughout the experiment.

*Hesp group*: Mice received 100 mg/kg Hesp orally^20^, every other day, all over the experiment.

EAC group: Mice received an intraperitoneal injection of 0.2 mL containing 2.5 × 10^6^ EAC cells per mouse on day “zero”^8^.

*Hesp-protected group*: Mice received 100 mg/kg Hesp orally, every other day for 2 weeks prior to EAC inoculation. Three days post inoculation; mice received 100 mg/kg Hesp orally, every other day, until the end of the experiment.

*Hesp-treated group*: Mice received distilled water orally, every other day, for two weeks prior to EAC inoculation. Three days after post-inoculation, mice received 100 mg/kg Hesp orally, every other day, until the end of the experiment.

*Cis-treated group*: Mice received distilled water orally, every other day, for two weeks prior to EAC inoculation. Three days post-inoculation; mice administrated a single intraperitoneal injection of Cis (5 mg/kg)^21^.

*Cis* + *Hesp-treated group*: Mice received distilled water orally, every other day, for two weeks prior to EAC inoculation. Three days post-inoculation; mice received a single intraperitoneal injection of Cis (5 mg/kg), concomitantly with 100 mg/kg Hesp orally, every other day, until the end of the experiment.

### Sampling

By the end of the experiment (on 28^th^ day post-inoculation), all animals were fasted and given isoflurane inhalation anesthesia for samples collection. Blood samples were gently drawn from the retro-orbital venous plexus into blank tubes without anticoagulant and centrifuged for 15 min at 3000 rpm for serum samples collection. The obtained serum samples were preserved at − 20 °C for the subsequent biochemical assessments.

After blood samples collection, mice were euthanized by cervical dislocation, and livers were instantly collected. Each liver was divided into two portions, one portion was rinsed in cold normal saline solution, dried using filter paper and then reserved at − 80 °C for further assessment of oxidant/antioxidant markers in hepatic tissues. The other portion was immediately kept in 10% neutral-buffered formalin for the histopathological and immunohistochemical examinations.

### Assessment of serum biochemical parameters

The serum total protein (CAT.NO. 310 001), albumin (CAT.NO. 210 001), cholesterol (CAT.NO. 230 001), and glucose (CAT.NO. 250 001) were measured following the colorimetric method of Tietz^22^; Tietz^23^; Young^24^ and Tietz^25^, respectively. Enzymatic activities of serum ALT (CAT.NO. 264 001), AST (CAT.NO. 260 001) and LDH (CAT.NO. 279 001) were determined following the protocols of Young^24^ while, the activities of amylase and lipase were measured according to Young^26^. Serum concentrations of AFP (CAT.NO. 30413) were estimated by an automated quantitative enzyme-linked fluorescent assay (ELFA) with mini-VIDAS®AFP according to Jang^27^.

### Assessment of hepatic oxidant/antioxidant biomarkers

Hepatic MDA (CAT.NO. MD 25 29) level, GSH (CAT.NO. GR 25 11) concentration, and CAT (CAT.NO. CA 25 17) activity were analyzed using colorimetric methods of Ohkawa et al.^28^, Beutler et al.^29^ and Aebi^30^, respectively.

### Histopathological investigation

The formalin-preserved liver specimens were trimmed, routinely processed, stained with Hematoxylin and Eosin following the method described by Bancroft and Gamble^31^. Semi-quantitation for hepatic lesions was evaluated based on the degree of severity as follow: (0) no changes, (1) mild (< 30% changes), (2) moderate (30–50%), and (3) severe (> 50% severe change)^32^.

### Immunohistochemical examination

Immunohistochemical investigation was conducted following the procedure illustrated by Abd El-Maksoud et al.^33^. The quantitative immune-reaction of Ki-67 and Caspase-3 were examined in five liver sections Zaki et al.^34^. Immuno-reaction in ten microscopic fields/each section was examined under high-power microscopical field (× 400). The percentage of positively-stained liver cells was examined by color deconvolution image J 1.52 p software (Wayne Rasband, National Institutes of Health (U.S.A.)).

### Statistical analysis

Data were displayed as mean values ± SE. The statistical (*p* < 0.05) significance among the different treated groups were proceeded using one-way ANOVA followed by Duncan’s post hoc.

## Results

### Hesperidin and/or cisplatin modulated serum biochemical alterations in EAC-bearing mice

Data presented in Table 1, clarified that there were significant (*P* < 0.05) declines in serum levels of total protein and albumin in EAC-bearing mice when compared to the control group. Nevertheless, the protection of EAC-bearing mice with Hesp or their treatment with Hesp and/or Cis significantly increased the corresponding values when compared to the EAC group to attain their normal control levels in both the Hesp-protected and Cis + Hesp-treated groups. On the other hand, evaluation of serum cholesterol and glucose concentrations did not implicate significant variations among the different experimental groups.

**Table 1:** Effects of EAC and/or different treatments on serum biochemical parameters in mice.

Parameters	Experimental groups						
	Control	Hesp	EAC	Hesp-protected	Hesp-treated	Cis-treated	Cis + Hesp-treated
Total protein (g/dl)	6.06 ± 0.24^a^	5.90 ± 0.22^a^	3.28 ± 0.20^c^	5.78 ± 0.26^a^	4.46 ± 0.39^b^	4.57 ± 0.23^b^	5.62 ± 0.25^a^
Albumin (g/dl)	4.24 ± 0.43^a^	4.14 ± 0.42^a^	1.62 ± 0.06^c^	3.92 ± 0.49^a^	2.66 ± 0.07^b^	2.84 ± 0.11^b^	3.83 ± 0.45^a^
Cholesterol (mg/dl)	82.00 ± 2.51	82.20 ± 2.89	91.20 ± 2.89	83.60 ± 2.79	87.00 ± 2.85	86.40 ± 2.90	85.80 ± 2.84
Glucose (mg/dl)	113.4 ± 2.84	112.6 ± 2.73	105.2 ± 2.58	110.4 ± 2.99	106.0 ± 2.17	107.8 ± 2.92	109.0 ± 2.97
ALT (U/L)	40.80 ± 1.53^c^	41.00 ± 1.95^c^	71.00 ± 1.76^a^	45.20 ± 2.06^c^	65.40 ± 2.80^a^	57.60 ± 2.11^b^	46.40 ± 1.75^c^
AST (U/L)	93.20 ± 1.85^c^	94.60 ± 2.25^c^	142.60 ± 2.94^a^	97.40 ± 2.56^c^	140.40 ± 1.29^a^	130.60 ± 2.34^b^	98.60 ± 2.89^c^
LDH (U/L)	731.4 ± 12.84^c^	727.4 ± 11.40^c^	885.8 ± 11.52^a^	739.8 ± 14.68^c^	824.8 ± 12.04^b^	766.6 ± 14.35^c^	755.2 ± 11.66^c^
Amylase (U/L)	161.4 ± 3.60^d^	160.2 ± 3.64^d^	649.8 ± 5.61^a^	169.6 ± 2.54^d^	369.6 ± 4.72^c^	402.0 ± 4.65^b^	170.4 ± 3.60^d^
Lipase (U/L)	41.40 ± 2.29^c^	42.60 ± 2.09^c^	170.40 ± 3.66^a^	50.80 ± 3.15^c^	148.60 ± 3.23^b^	156.40 ± 3.61^b^	51.00 ± 3.16^c^
AFP (ng/ml)	0.11 ± 0.01^c^	0.13 ± 0.01^c^	1.45 ± 0.11^a^	0.25 ± 0.02^c^	1.20 ± 0.09^b^	1.04 ± 0.07^b^	0.27 ± 0.02^c^

Values are means ± SE (n = 10). Different letters in the same row indicate significant differences at *p* < 0.05. EAC: Ehrlich ascites carcinoma; Hesp: hesperidin; Cis: cisplatin; ALT: alanine aminotransferase; AST: aspartate aminotransferase; LDH: lactic dehydrogenase; AFP: alpha-fetoprotein; MDA: malondialdehyde; TAC: total antioxidant capacity.

Compared with the control group, marked increases were noticed in the mean values of serum activities of ALT, AST, LDH, amylase and lipase in EAC-bearing mice. However, the administration of Hesp and/or Cis significantly reduced the corresponding values of these enzymes with retaining their normal control levels in the Hesp-protected and Cis + Hesp-treated groups (Table 1).

### Hesperidin and/or cisplatin reduced serum AFP levels in EAC-bearing mice

The results of serum tumor biomarkers as shown in Table 1 explored that serum AFP concentrations exhibited a significant (*P* < 0.05) increase in EAC-bearing mice compared with the control group. In the Hesp-protected and Cis-Hesp treated groups, the mean values of AFP were significantly lower than EAC group and were much closer to their relevant control levels.

### Hesperidin and/or cisplatin promoted the hepatic oxidant/antioxidant status in EAC-bearing mice

**Fig. 2:**
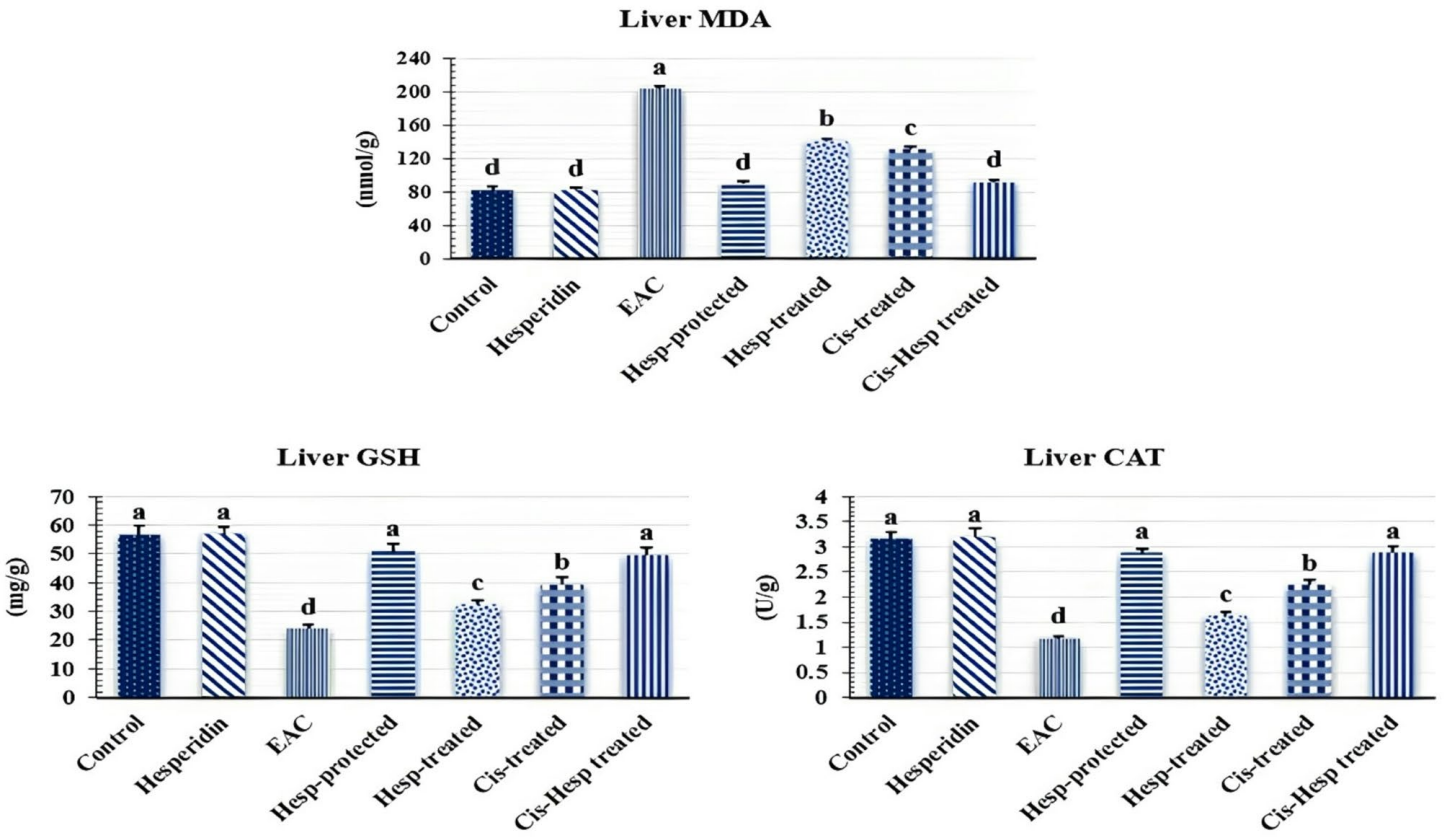
Effect of hesperidin and/or cisplatin on hepatic oxidant/antioxidant status in EAC-bearing mice (n = 7). Different letters indicate significant differences at *p* < 0.05. EAC: Ehrlich ascites carcinoma; Hesp: hesperidin; Cis: cisplatin; MDA: malondialdehyde; GSH: reduced glutathione; CAT: catalase.

As clarified in Fig. 2, no significant (*p* < 0.05) changes were observed in the mean values of hepatic oxidant/antioxidant markers between the Hesp and the control groups. However, mice inoculated with EAC demonstrated significant elevations in the hepatic MDA level, concurrently with significant declines in the hepatic GSH concentration and CAT activity when compared to the corresponding normal control values. On the other hand, the administration of Hesp and/or Cis significantly improved the EAC-induced impairments in the hepatic oxidant/antioxidant markers compared with EAC group, with restoring their normal control levels in the Hesp-protected and Cis + Hesp-treated groups.

### Hesperidin and/or cisplatin improved the hepatic histoarchitecture in EAC-bearing mice

**Fig. 3:**
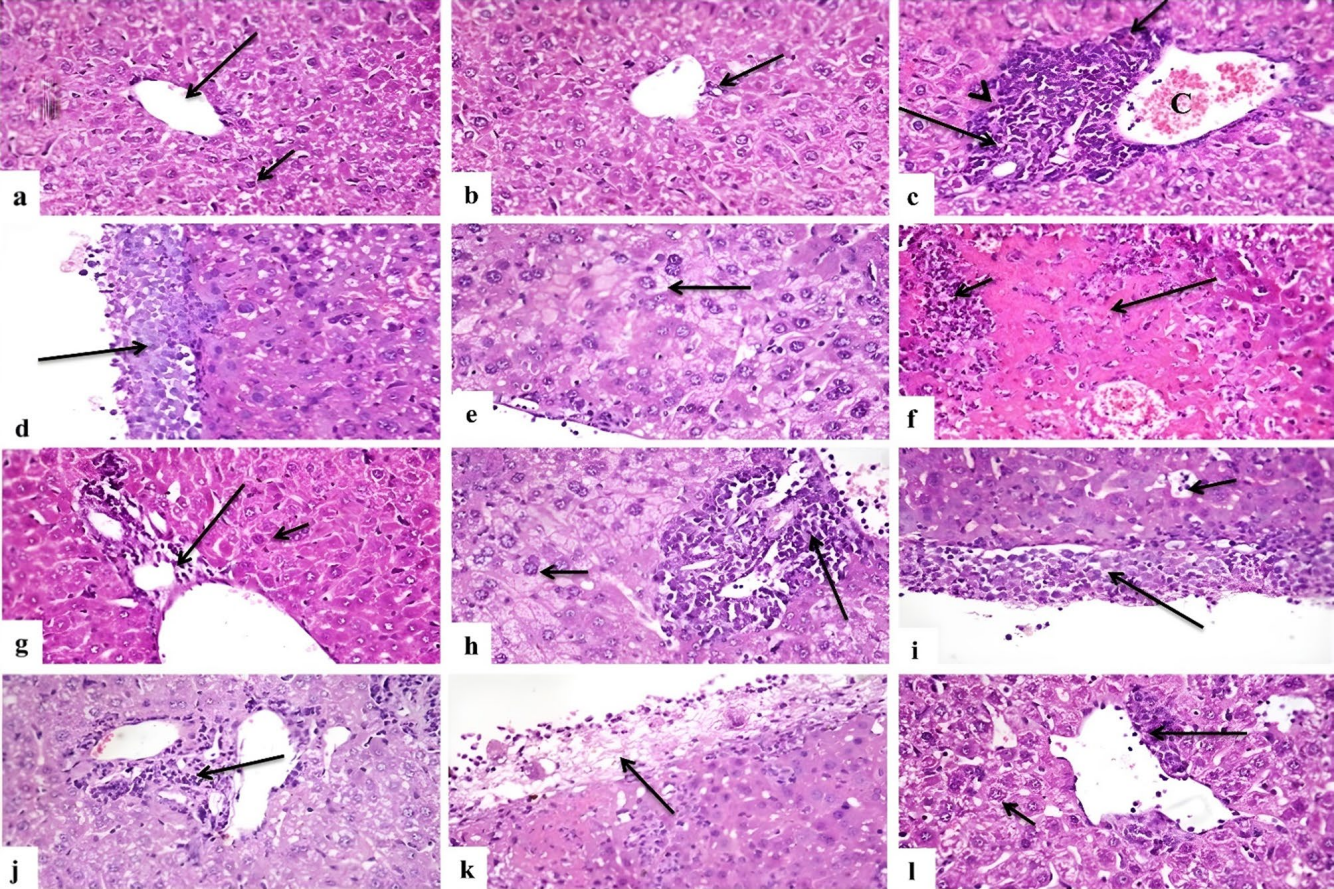
Photomicrographs of histopathological alterations in liver sections of different groups. (**a**) Control and (**b**) Hesp groups showing normal histological structure of central vein (long arrow) and hepatocytes (short arrow). (**c**) EAC group showing infiltration of portal area with tumor cells (long arrow), with mitosis (arrowhead) and tumor giant cell (short arrow), portal vessel congestion (**C**). (**d**) EAC group showing infiltration of hepatic capsule with tumor cells (arrow). (**e**) EAC group, vacuolar degeneration and karyocytomegaly of hepatocytes (arrow). (**f**) Hesp-protected group showing necrosis of tumor cells (long arrow) that infiltrated with mononuclear cells (short arrow). (**g**) Hesp-protected group, note infiltration of portal area with few tumor cells (long arrow) with mild vacuolar degeneration of hepatocytes (short arrow). (**h**) Hesp-treated group showing heavy infiltration of portal area with tumor cells (long arrow) with vacuolar degeneration of hepatocytes (short arrow). (**i**) Hesp-treated group showing heavy infiltration of hepatic capsule with tumor cells (long arrow), sinusoidal infiltration with neoplastic cells (short arrow). (**j**) Cis-treated group showing infiltration of portal area with few neoplastic cells (arrow). (**k**) Cis-treated group, few cancer cells infiltrating the hepatic capsule (arrow). (**l**) Cis + Hesp-treated group showing infiltration of portal area with few neoplastic cells (long arrow) with mild vacuolar degeneration of hepatocytes (short arrow) (H&EX400).

The histopathological changes in the liver of the different treated groups were presented in Table 2 and Fig. 3. Control (Fig. 3a) and Hesp groups (Fig. 3b) showed normal histological structure of liver. In EAC group, infiltration of neoplastic cells in portal areas (Fig. 3c) blood sinusoids and hepatic capsule (Fig. 3d), some tumor foci also present replacing hepatic parenchyma, these foci of tumor cells characterized by nuclear hyperchromacia, presence of multinucleated tumor giant cells with frequent mitotic figures, portal blood vessels and blood sinusoids were congested, hepatocytes showed vacuolar degeneration with karyocytomegaly in most cells (Fig. 3e). Hesp-protected group revealed areas of necrosis of tumor cells that infiltrated with mononuclear cells (Fig. 3f), neoplastic cells that infiltrate portal areas, blood sinusoids and hepatic capsule were fewer or even absent (Fig. 3g), portal blood vessels were congested, mild vacuolar degeneration of hepatocytes, few hepatocytes showed mild karyocytomegaly. Hesp-treated group showed heavy infiltration of neoplastic cells in portal areas (Fig. 3h), blood sinusoids and hepatic capsule and also foci in liver parenchyma (Fig. 3i), portal blood vessels and blood sinusoids were congested, hepatocytes revealed severe vacuolar degeneration with karyocytomegaly in most cells. Cis-treated group revealed fewer or even no tumor cells in portal areas (Fig. 3j), blood sinusoids, hepatic capsule and parenchyma, there was necrosis of some tumor cells in hepatic capsule (Fig. 3k) and parenchyma, there was severe vacuolar degeneration with karyocytomegaly in most hepatocytes. Cis + Hesp-treated group showed smaller foci of parenchymal tumor cells with necrosis of some of them, portal areas and hepatic capsule were infiltrated with few neoplastic cells, with congestion of portal blood vessels and blood sinusoids, there was mild vacuolar degeneration and karyocytomegaly of hepatocytes (Fig. 3l).

**Table 2:** Scoring of histopathological alterations in liver of EAC-inoculated as well as the different treated groups.

Lesion	Experimental groups						
	Control	Hesp	EAC	Hesp-protected	Hesp-treated	Cis-treated	Cis + Hesp-treated
Tumor cells infiltrating portal areas	0	0	3	1	3	1	1
Tumor cells infiltrating sinusoids	0	0	3	1	3	1	1
Tumor cells infiltrating capsule	0	0	3	1	3	1	1
Tumor cells infiltrating parenchyma	0	0	3	1	3	1	1
Congestion of portal blood vessels	0	0	3	1	3	3	2
Sinusoidal congestion	0	0	3	1	3	3	2
Vacuolar degeneration of hepatocytes	0	0	3	1	3	3	1
Karyocytomegaly of hepatocytes	0	0	3	1	3	3	1

The score system was designed as: score 0 = absence of the lesion in all mice of the group (n = 5), score 1 = (< 30%), score 2 = (< 30–50%), score 3 = (> 50%). EAC: Ehrlich Ascites Carcinoma, Hesp: hesperidin, Cis: cisplatin.

### Hesperidin and/or Cisplatin downregulated Ki-67 and upregulated caspase-3 proteins expressions of the neoplastic cells in hepatocytes of EAC-bearing mice

**Fig. 4:**
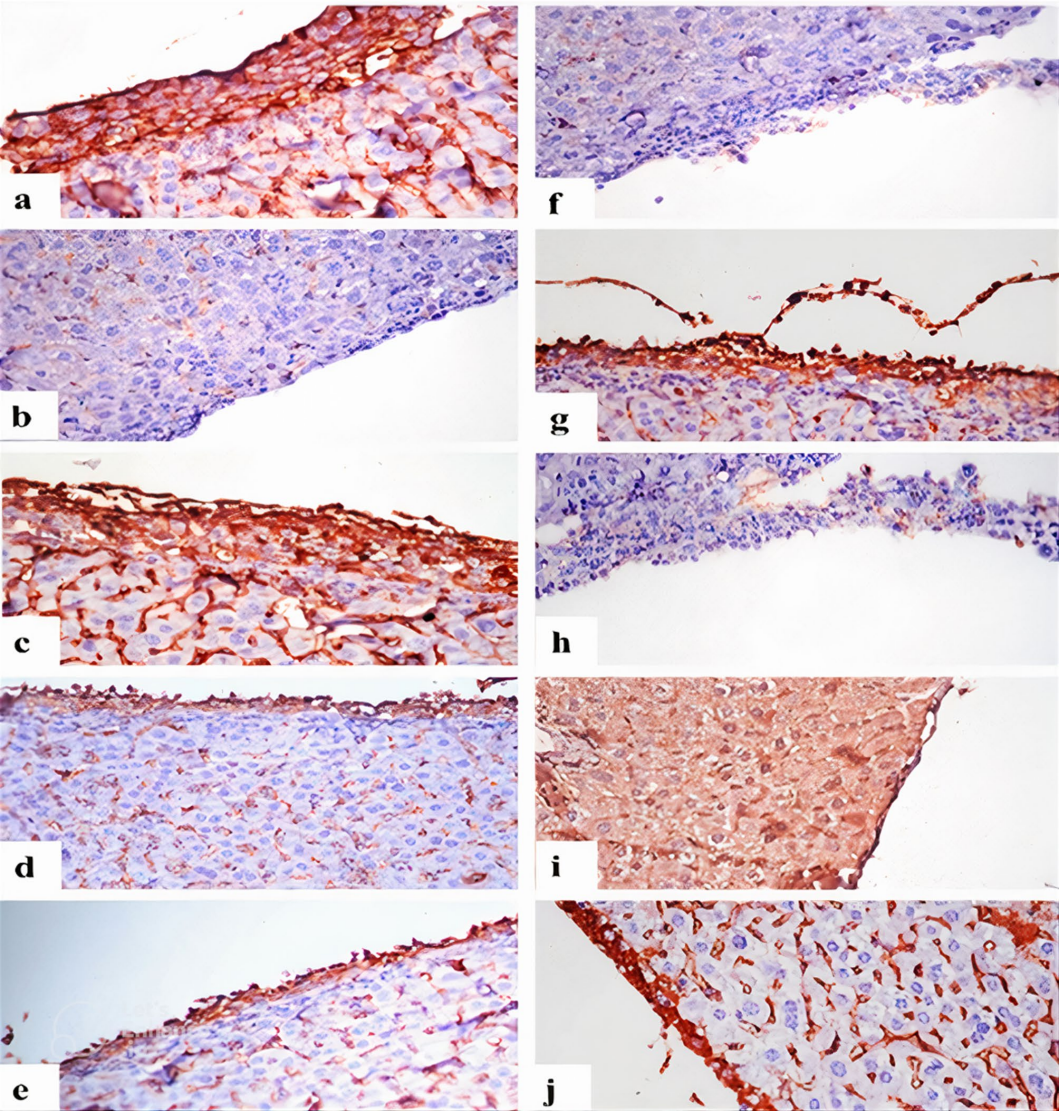
Immunostaining of Ki-67 and caspase-3 in hepatic capsule. (**a**) EAC group showing strong expression of Ki-67 in neoplastic cells infiltrating hepatic capsule. (**b**) Hesp-protected group showing weak or nil expression of Ki-67 in few neoplastic cells. (**c**) Hesp-treated group showing strong positive expression of Ki-67 in neoplastic cells in hepatic capsule. (**d**) Cis-treated group showing expression of Ki-67 in few neoplastic cells infiltrating hepatic capsule. (**e**) Cis + Hesp-treated group showing expression of Ki-67 in in few neoplastic cells. (**f**) EAC group showing weak expression of caspase-3 in neoplastic cells infiltrating hepatic capsule. (**g**) Hesp-protected group showing strong expression of caspase-3 in neoplastic cells. (**h**) Hesp-treated group showing weak expression of caspase-3 in neoplastic cells infiltrating hepatic capsule. (**i**) Cis-treated group showing strong expression of caspase-3 in hepatic capsule. (**j**) Cis + Hesp-treated group showing strong expression of caspase-3 in hepatic capsule (Ki-67 and caspase-3 X400).

**Fig. 5:**
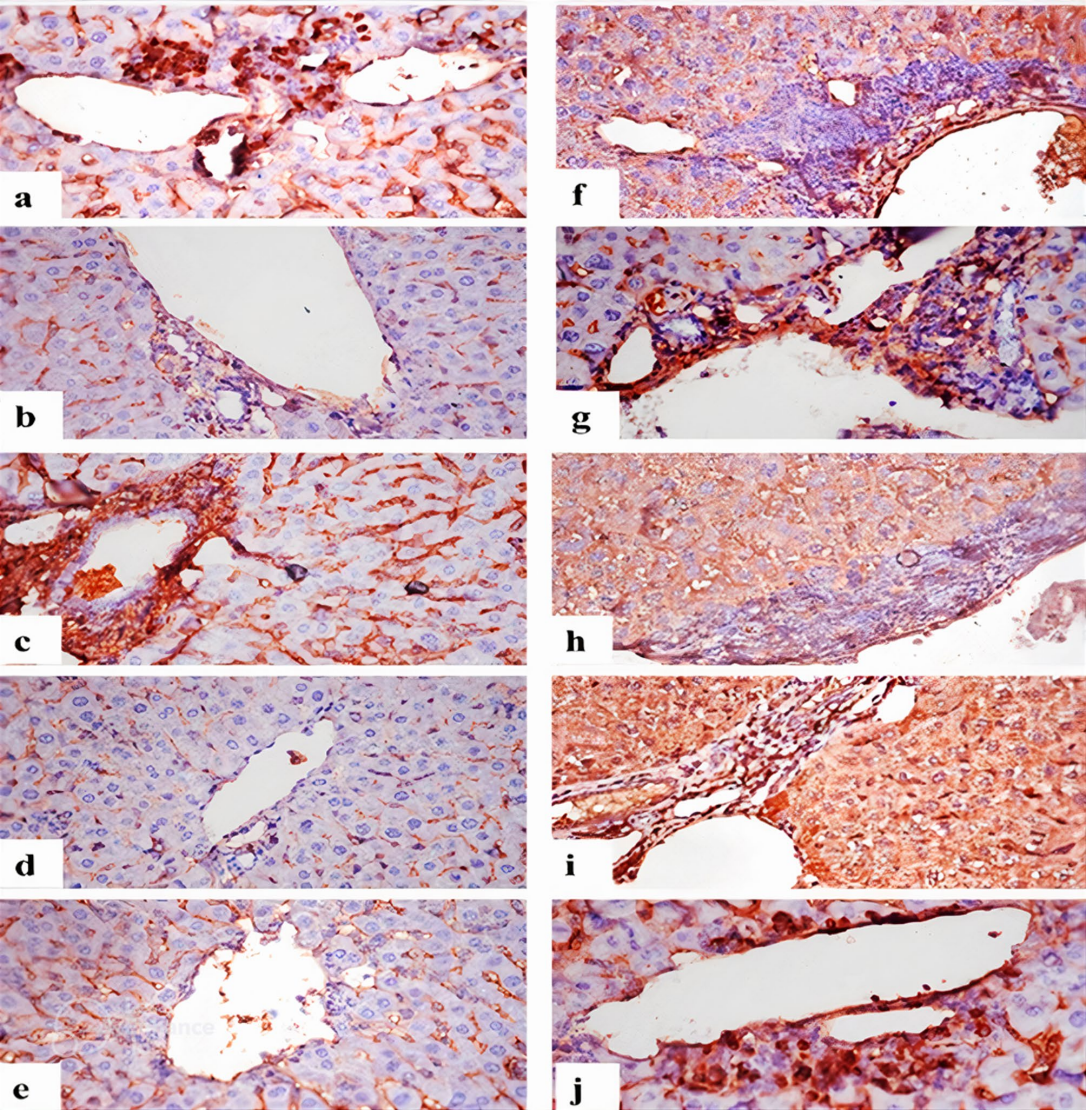
Immunostaining of Ki-67 and caspase-3 in portal areas. (**a**) EAC group showing strong expression of Ki-67 in neoplastic cells infiltrating portal area. (**b**) Hesp-protected group showing weak expression of Ki-67 in neoplastic cells infiltrating portal area. (**c**) Hesp-treated group showing strong expression of Ki-67 in neoplastic cells infiltrating portal area. (**d**) Cis-treated group showing weak expression of Ki-67 in neoplastic cells infiltrating portal area. (**e**) Cis + Hesp-treated group showing no immune expression of Ki-67 in portal area. (**f**) EAC group showing weak expression of caspase-3 in neoplastic cells infiltrating portal area. (**g**) Hesp-protected group showing strong expression of caspase-3 in neoplastic cells infiltrating portal area. (**h**) Hesp-treated group showing weak expression of caspase-3 in neoplastic cells infiltrating portal area. (**i**) Cis-treated group showing strong expression of caspase-3 in neoplastic cells infiltrating portal area. (**j**) Cis + Hesp-treated group showing strong expression of caspase-3 in neoplastic cells infiltrating portal area (Ki-67 and caspase-3 X400).

Immunostaining reactivity of Ki-67 and caspase-3 (Area %) in hepatic tissue of different experimental groups was presented in Table 3 and Figs. 4,5. The EAC group exhibited strong Ki-67 expressions in neoplastic cells and hepatocytes (Figs. 4a and 5a), while for caspase-3 there were no immune-reactive neoplastic cells (Figs. 4f and 5f). Hesp-protected group showed weak Ki-67 expressions in neoplastic cells and hepatocytes (Figs. 4b and 5b), while showed strong caspase-3 expressions in neoplastic cells and weak caspase-3 expressions in hepatocytes (Figs. 4g and 5g). Hesp-treated group showed strong positive Ki-67 expressions in neoplastic cells and hepatocytes (Figs. 4c and 5c), while no immune-reactive neoplastic cells with strong expressions in the hepatocytes were observed with caspase-3 immunreactivity (Figs. 4h and 5h). Cis-treated group displayed weak Ki-67 expressions in neoplastic cells and hepatocytes (Figs. 4d and 5d) with strong caspase-3 expressions in both neoplastic cells and hepatocytes (Figs. 4i and 5i). Cis + Hesp-treated group showed weak Ki-67expressions in both neoplastic cells and hepatocytes (Figs. 4e and 5e) with strong caspase-3 expressions in neoplastic cells and weak expressions in hepatocytes (Figs. 4j and 5j).

**Table 3:** Area % of Ki-67 and caspase-3 expression in hepatocytes of different experimental groups.

Parameters	Experimental groups				
	EAC	Hesp-protected	Hesp-treated	Cis-treated	Cis + Hesp-treated
Ki-67%	42.2 ± 2.4^a^	13.2 ± 1.9^b^	46.8 ± 4.8^a^	16.3 ± 3.2^b^	16.9 ± 2.9^b^
Caspase-3%	58.3 ± 7.1^a^	23.9 ± 1.2^b^	49.9 ± 6.3^a^	53.9 ± 3.9^a^	19.8 ± 5.2^b^

Values are expressed as means ± SE, (n = 5). Different letters in the same row indicate significant differences at *p* < 0.05. EAC: Ehrlich Ascites Carcinoma, Hesp: hesperidin, Cis: cisplatin.

## Discussion

**Fig. 6:**
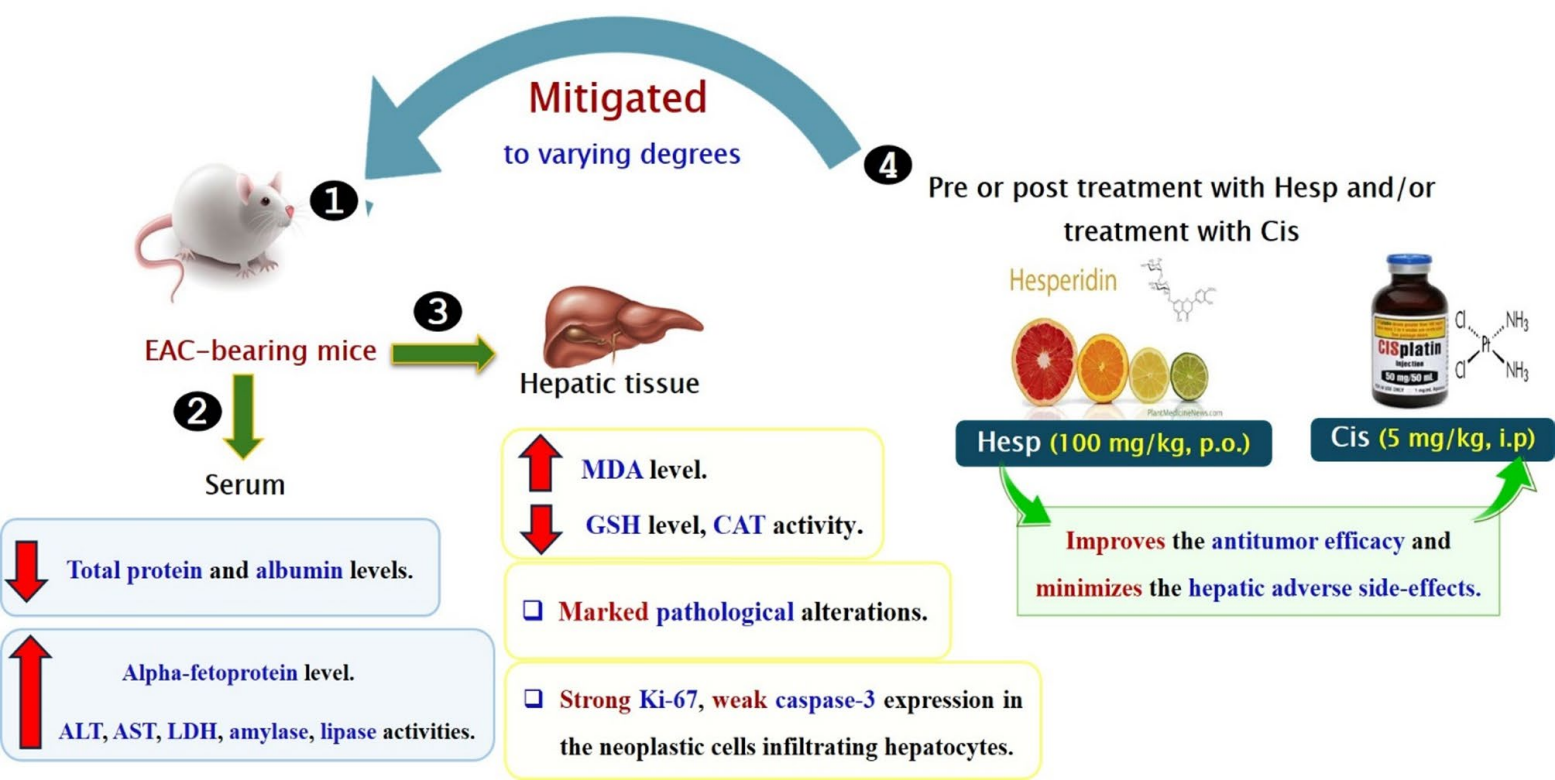
A diagram summerizing the effect of administration of hesperidin and/or cisplatin on the hepatocytotoxicity of Ehrlich ascites carcinoma in mice.

Chemotherapy is commonly used to kill cancer cells and thus can treat various types of cancer effectively. But unfortunately, as the chemo drug passes throughout the body, it can kill and damage also the healthy cells resulting in a lot of side-effects rendering the effectiveness and side-effects of chemotherapy a problematic issue for many cancer patients^35^. Nowadays, great attention has been directed toward searching for novel therapeutic strategies for cancer treatment depending on the use of agents with a higher anticancer potency and lower adverse side-effects in comparison with the chemotherapeutic drugs^36^. Hesperidin, an active flavanone glycoside of citrus fruits, has been proven to have antioxidant and anti-inflammatory properties as well as anticancer effects^35,37^. Therefore, the present study aimed to assess the potential protective and therapeutic anticancer effects of hesperidin as a natural product when used alone or in combination with cisplatin, a well-known chemotherapeutic drug, against EAC in mice with greatest emphasis on the hepatoprotective actions of hesperidin as summarized in Fig. 6.

Ehrlich ascites carcinoma is a transplantable tumor model in mice that has been extensively used in cancer research. While EAC itself is primarily an intraperitoneal tumor, studies have demonstrated its potential to metastasize to various organs, including the liver^38^. Furthermore, in some investigations, mice with EAC tumors had higher levels of liver enzymes which are implicated in the etiology of liver inflammation, as well as biomarkers such as tumor necrosis factor α, α fetoprotein, and caspase-3, Bcl2, and DNA damage highlighting the relevance of EAC to liver metastases^39^. These findings underscore the importance of the EAC model in studying liver metastases and evaluating potential therapeutic interventions targeting liver involvement in cancer progression.

The current serum biochemical analysis of EAC-bearing mice revealed marked reductions in the levels of total protein and albumin which might be attributed to decreased protein synthesis in the liver as a result of hepatic injury. This explanation was further supported in the current research by the significant elevation in the serum enzymatic activities of ALT and AST in EAC-bearing mice which might be related to drastic conditions caused by the toxic activity of tumor cells in the liver and leakage of these enzymes from damaged hepatocytes or increase hepatic cells permeability^40,41^. The elevation in enzymatic activities of amylase and lipase could be the result of pancreatic duct injury or obstruction due to pancreatitis or pancreatic trauma^42^ while the significant increase in the activity of LDH could be attributed to the role of EAC in enhancing glycolysis during the growth of tumor^43^. Interestingly, the administration of Hesp as a protective agent against EAC or as a concomitant treatment with Cis considerably improved the alterations observed in these biochemical variables compared to groups treated with Hesp or Cis alone. These findings agree with the findings of Abdallah et al.^44^, who found that hesperidin supplementation improved the changes in total protein and albumin levels produced by acrylonitrile-induced toxicity in rats. Consistently, Hozayen et al.^45^ reported that the pre-treatment of EAC with hesperidin successfully ameliorated the elevated serum ALT and AST activities suggesting a hepatoprotective role for Hesp. Further, Donia et al.^43^ have proven the protective effect of Hesp against pancreatic injuries caused by EAC or cisplatin which was explained by its antitumor and antioxidant activities that protect proteins from free radicals formed by EAC cells and cisplatin.

In this study, serum glucose and cholesterol concentrations did not show significant variations among the different experimental groups. While EAC may influence certain metabolic pathways, there is limited evidence to indicate significant effects on blood sugar and cholesterol levels in the previous studies. There is no particular explanation, but we suggest that the body may activate other compensatory mechanisms, to maintain normal serum levels of glucose and cholesterol despite the tumor’s metabolic demands. For example, the increased consumption of glucose by cancer cells via glycolysis may be counteracted by increased endogenous glucocorticoids production as a result of the stress of cancer leading to increased blood glucose and thus maintaining normal serum glucose levels^46–48^. The interference of cholesterol excretion due to liver involvement in the course of cancer may compensate for the tumor’s cholesterol consumption and maintain normal cholesterol concentrations^49^. Further, the animal’s inflammatory and immune responses to the tumor may influence glucose and lipid metabolism, preventing changes in their serum levels.

Alpha-fetoprotein is a tumor marker expressed by AFP gene and is considered the gold standard tumor marker for hepatocellular carcinoma (HCC). Its expression was found to be upregulated during hepatocarcinogenesis, liver metastases, and benign liver disorders^50^. The elevation in serum levels of AFP in this study might be due to liver metastasis resulted from EAC, a finding that agreed with Medhat et al.^51^ and Aldubayan et al.^52^. The treatment of EAC-bearing mice with a combination of Hesp and Cis significantly counteracted EAC-induced increase in serum concentration of AFP providing that these protocols exhibit significant antitumor activity compared to the Hesp or Cis-treated groups. The anticancer potential of Hesp either alone or combined with other drugs has been demonstrated in previous studies under both in vitro and in vivo conditions^37^. The chemoprevention of Hesp in azoxymethane-induced colon carcinogenesis in rats has been also established^53^. Moreover, rats treated with orange and mandarin juice had a 22% decline in colon cancer and a 29% reduction in lung cancer, and these effects were attributed to the high contents of flavonoids such as Hesp in these juices^54^. The potential therapeutic strategy combining citrus bioflavonoids with traditional heterocyclic medications has also been suggested to treat brain cancer^55^.

Regarding the mechanisms lying behind the anticancer effect of Hesp, the anticarcinogenic activities of Hesp have been proved to be through its modulating effects on the allover hallmarks of cancer, including downregulating the pro-inflammatory mediators and enzymes, enhancing antioxidant defense system and preventing cancer cell proliferation by augmenting apoptosis in cancer cells^18^. Previous research has been focused on the role of antioxidants for the treatment of numerous diseases, including cancers, particularly those of natural origin, due to their higher therapeutic potency and lower adverse side-effects than synthetic antioxidants^56^. Further, the antitumor activity of some plant-derived extracts was attributed to its antioxidant contents which could exert direct cytotoxic action against tumor cells such as EAC in experimental animals^57,58^.

Herein, EAC-bearing mice demonstrated significant increments in hepatic tissue concentrations of MDA accompanied by marked decrease in the hepatic GSH concentration and CAT activity. These findings agree with the previous reports of the existence of oxidative stress caused by tumor cells that results from accumulation of free radicles and the higher consumption of antioxidative enzymes^59,60^. However, our data showed that Hesp effectively ameliorated the alterations in the hepatic oxidant-antioxidant markers, particularly when it was given for protection against EAC or when it was co-administrated with Cis for treatment of EAC-bearing mice supporting that the reported anticancer effects of Hesp could be associated with its antioxidant activities^43^. Choi^61^ reported that Hesp effectively decreased protein oxidation in 7,12-dimethylbenz(a)anthracene (DMBA)-treated mice, which was known to induce oxidative hepatic and mammary glands damage.

At the molecular level, Hesp was documented to induce its anticancer effects by interacting with numerous recognized cellular targets, thus inhibiting the growth and proliferation of cancer cells via apoptosis induction and cell cycle arrest^37^. To explore this effect in the present study, the liver tissue was tested for Ki-67 and caspase-3 expressions. The Ki-67 gene, a well-known cell proliferation marker, exists only during active phases of the cell cycle. However, caspase-3 is essential in the cell apoptosis execution phase, and thus it can be measured to confirm the apoptotic cell death^62^. In this work, EAC-bearing mice demonstrated a strong Ki-67 expression and a weak caspase-3 expression in tumor cell, which was compatible with the findings of Mohammed et al.^63^ and Hashem et al.^64^. Protection of mice with Hesp or its co-administration with Cis in our study modulated EAC-induced alterations in Ki-67 and Caspase-3 expressions in hepatic tissues. The strong caspase-3 expressions of neoplastic cells in Hesp-protected and Cis-Hesp-treated mice confirms the induction of apoptosis while the decreased expression of Ki-67 can be considered a sign of tumor regression that contributes to the anticancer effects of these treatments^65^. Studies on colon cancer have suggested that Hesp stimulates specific intracellular death-receptor pathways in colon cancer cells by causing DNA fragmentation and the formation of perinuclear apoptotic bodies, and this apoptosis was induced typically via the up-regulation of Caspase-3 and Bax^66,67^. In addition to caspases, Hesp was also found to target Bax and Bcl-2 for the induction of apoptosis and COX-2, MMP-2, and MMP-9 for the prevention of angiogenesis and metastasis^37^. Banjerdpongchai et al.^35^ provided another confirmation when Hesp was found to be effective against hepatocellular carcinoma by inducing apoptosis of human HepG2 cells via mitochondrial and death receptor pathways. The results of immunohistochemistry came into line with the recorded histopathological changes of hepatic tissue which revealed that hesperidin greatly improved the alterations observed in hepatic histoarchitecture due to EAC induction either when used for protection or combined with Cis as a treatment.

Regarding Cis treatment, the presented data clarified that treatment of EAC-bearing mice with Cis did not result in obvious improvement in the hepatic oxidant-antioxidant markers whereas their values were significantly different from control levels compared to Hesp-protected and Cis + Hesp-treated groups. Additionally, treatment of EAC bearing mice with Cis did not result in remarkable improvement in the selected hepatic serum biomarkers compared to Hesp-protected and Cis + Hesp-treated groups. These findings were also supported by the recorded histopathological observations indicating a hepatotoxic effect for Cis. Hepatotoxicity was a dose-limiting side-effect associated with Cis-based chemotherapy as reported in previous studies, mainly due to generation of various ROS and induction of oxidative stress ^68–70^. Interestingly, in this study, the addition of Hesp to Cis as a combination therapy for EAC greatly alleviated Cis-induced functional and histopathological hepatic damage without lowering its potential cytotoxic effect on cancer cells, suggesting Hesp as a potent anticancer agent protecting the liver against the Cis-hepatotoxic effects^68^ that may be attributed to the ability of Hesp to arrest tumor growth via the apoptotic pathway by downregulating the expression of Bcl-2, an antiapoptotic gene, and upregulating the expression of the Caspase3 and Bax genes, apoptotic genes^43^ as well as its ability to prevent oxidative damage^59^.

## Conclusion

The data obtained from this study indicated that administration of Hesp and/or Cis could ameliorate the hepatotoxic effects of EAC. In addition, Hesp minimized the harmful hepatic adverse side-effects induced by the treatment with Cis without affecting its potential cytotoxic effect on cancer cells. Thus, our findings recommend hesperidin to be effectively supplemented with other chemotherapeutic drugs like Cis for enhancing its antitumor efficacy and decreasing its hepatotoxic effects. Nevertheless, further pre-clinical and clinical studies are warranted to increase the translation applicability of Hesp for example, by applying nanotechnology-based strategies to enhance the therapeutic benefits of hesperidin and improve its delivery, bioavailability, and efficacy to confirm the full potential of this bioflavonoid in cancer prevention and intervention.

## Data Availability

The authors confirm that all data supporting the finding of this study are available within this published article.
